# A 73-Year-Old Man with Long-Term Immobility Presenting with Abdominal Pain

**DOI:** 10.1371/journal.pmed.1000092

**Published:** 2009-07-14

**Authors:** Salomone Di Saverio, Gregorio Tugnoli, Paolo Emilio Orlandi, Marco Casali, Fausto Catena, Andrea Biscardi, Omeshnie Pillay, Franco Baldoni

**Affiliations:** 1 Departments of Emergency and Surgery, Emergency Surgery and Trauma Surgery Unit, Trauma Center, Maggiore Hospital–Bologna Local Health District, Bologna, Italy; 2 Department of Radiology, Maggiore Hospital–Bologna Local Health District, Bologna, Italy; 3 Department of Emergency and Transplant Surgery, S. Orsola Malpighi University Hospital, Bologna, Italy; 4 Addington Hospital, Durban, KwaZulu-Natal, South Africa; Chinese University of Hong Kong, China

## Abstract

Salomone Di Saverio and colleagues discuss the diagnosis and management of a man presenting with symptoms of partial intestinal obstruction.

In summer 2008, a 73-year-old man arrived in the emergency room with vague abdominal pain. On examination, his abdomen was distended and tympanic, but soft and non-tender on palpation. The patient had been immobile and bed-ridden since 1995, when he had neurosurgical resection of a medullary ependymoma of the spine, with residual paraplegia, peripheral neuropathy, and a neuropathic bladder with secondary chronic renal failure. The patient had a medical history of hypertension and type II diabetes mellitus and suffered from chronic constipation with recurrent episodes of partial bowel obstruction.

Given his past history, the current presentation was in keeping with a further episode of bowel obstruction. At this stage, we needed to assess the degree of obstruction (complete or partial) and its possible causes.

## What Investigations Are Required in Assessing Bowel Obstruction?

**Figure 1:**
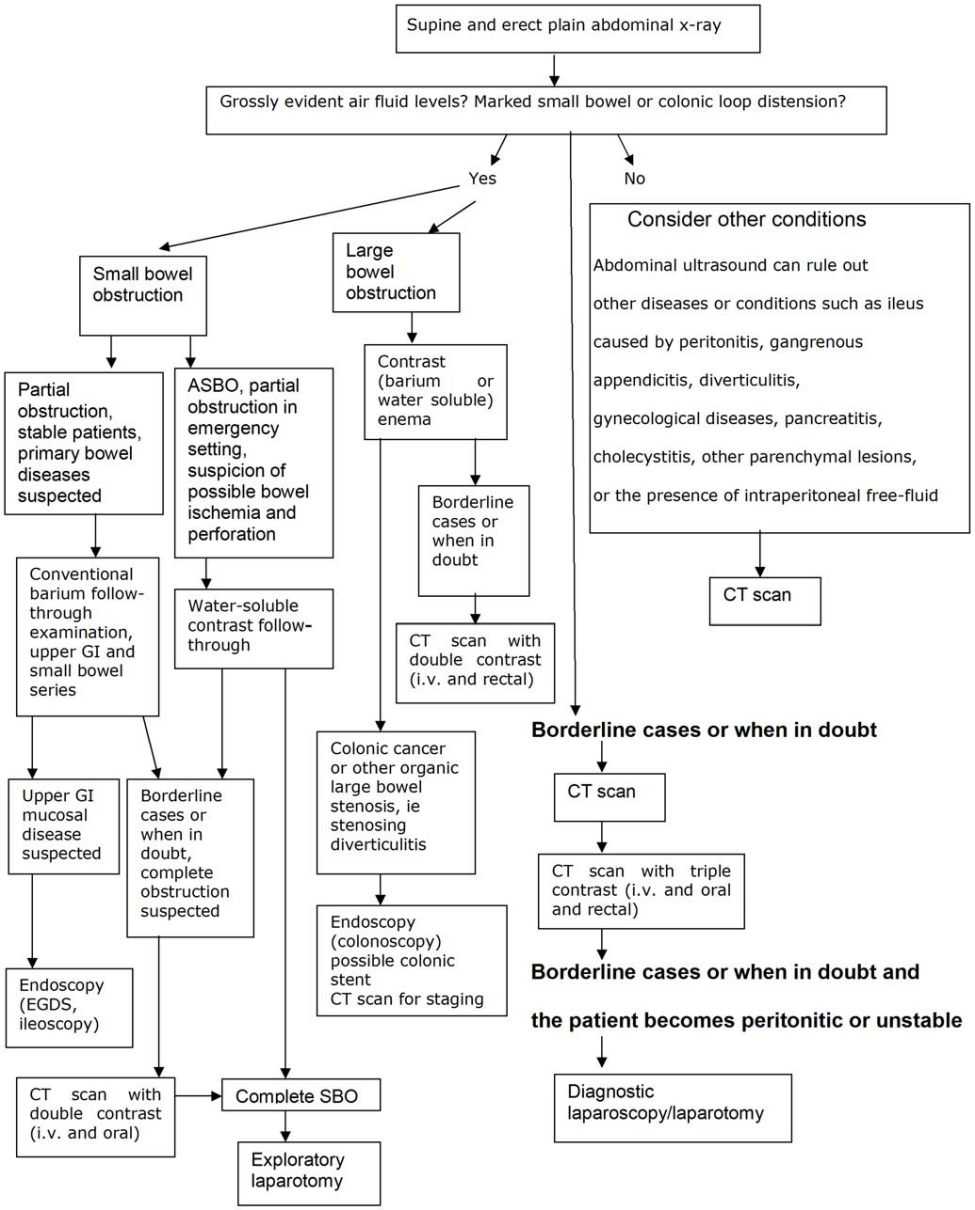
Flow diagram for diagnostic assessment of bowel obstruction. ASBO, adhesive small bowel obstruction; EGDS, esophagogastroduodenoscopy; GI: gastrointestinal; i.v., intravenous; SBO, small bowel obstruction.

The full diagnostic workup is shown in Box 1, and Figure 1 shows a flow diagram for diagnostic assessment of bowel obstruction. The usual sequence of investigations starts with plain abdominal X-ray (see Figure 1). In the absence of grossly distended bowel loops on X-ray, and if the patient's condition is stable, ultrasound can be useful to rule out (1) other conditions or diseases causing paralytic ileus or (2) the presence of intraperitoneal free fluid. If the plain abdominal X-ray shows air fluid levels and grossly distended bowel loops, the level and possible site of obstruction must be assessed (i.e., small bowel or large bowel), and the further diagnostic workup proceeds accordingly. When in doubt, or when the clinical and radiological findings are not clear enough to suggest the best further diagnostic steps, abdominal computed tomography (CT) scan may be helpful, with the eventual adjunct of triple contrast (intravenous, oral, and/or rectal). The last diagnostic options are diagnostic laparoscopy or exploratory laparotomy.

**Box 1. Intestinal Obstruction: Diagnostic Workup**

- Supine and erect plain abdominal X-ray
- Abdominal ultrasound (this is of limited value in bowel obstruction and/or in patients with distended bowel because the air, limiting ultrasound transmission, may obscure and camouflage the underlying findings)
- CT scan
- Conventional barium follow-through examination, upper gastrointestinal and small bowel series, enteroclysis (fluoroscopic X-ray of the small intestine) [18]
- Water-soluble contrast follow-through (this investigation is safer than barium in cases of perforation and peritoneal spread and has possible therapeutic value in the case of adhesive small intestine obstruction [19])
- Contrast (barium or water-soluble) enema
- CT scan with double contrast (intravenous and oral or rectal)
- Magnetic resonance imaging
- Endoscopy (colonoscopy, esophagogastroduodenoscopy, ileoscopy)
- Laparoscopy

**Figure 2:**
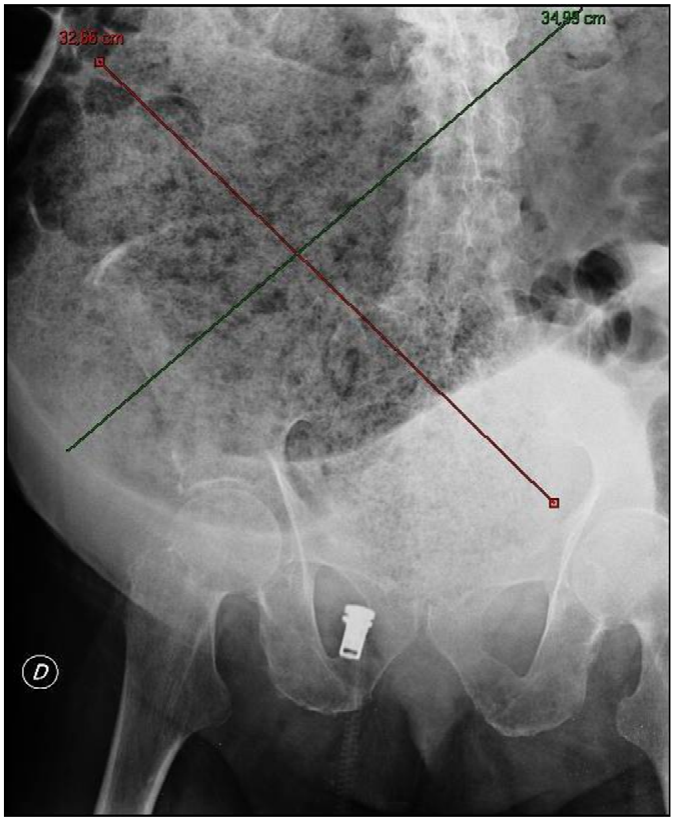
Initial plain abdominal X-ray done in emergency room showing a huge faecal impaction extending from the pelvis upwards to the left subphrenic space and from the left towards the right flank, measuring over 40 cm in length and 33 cm in width.

Plain abdominal X-ray was ordered. This showed a huge faecal impaction extending from the pelvis upwards to the left subphrenic space and from the left towards the right flank, measuring over 40 cm in length and 33 cm in width (Figure 2). There was also massive dilatation of the descending and sigmoid colon.

## At This Stage, What Was Our Differential Diagnosis?

This patient has partial large bowel obstruction. The differential diagnosis is between malignant obstructing diseases, such as colon cancer, or benign conditions, such as sigmoid volvulus or diverticulitis. These benign conditions can be life-threatening because of the risk of colonic ischaemia and/or perforation.

Given the history of recurrent chronic constipation, in a chronically bed-ridden patient with previous spinal surgery and residual paraplegia, we thought it was likely that this patient had faecal impaction *without* an anatomical lesion causing the impaction. Pinpointing the exact cause of the constipation in this patient was difficult, as he had a complex medical history and presentation—renal failure, dehydration, electrolyte imbalance, diabetes mellitus, and peripheral neuropathy and paraplegia secondary to spinal cord tumour, in an immobile bed-ridden patient with mild depression. Common causes of constipation are shown in Box 2. Some of the causal and contributing factors for constipation in patients undergoing palliative care are shown in Box 3.

**Box 2. Causes of Constipation**

- **Endocrinological**: diabetes mellitus, hypopituitarism, hypothyroidism, pseudohypoparathyroidism, hypocalcaemia, phaeochromocytoma, glucagonoma, pregnancy
- **Metabolic**: uraemia, hypokalaemia, porphyria, amyloidosis, dehydration
- **Neurological**: Parkinson disease, brain tumour, multiple sclerosis, sclerodermia, spinal cord injuries, tumours
- **Psychiatric**: depression, psychosis, anorexia nervosa, obsessive-compulsive disorders
- **Operations**: pelvic operations, anal operations, narrowing following anastomoses
- **Organic obstructive diseases**: tumours, adherences, strangulated hernias, volvulus, invagination, endometriosis
- **Diet**: inadequate intake of fibre or fluids
- **Lifestyle changes**: immobility, vacation
- **Functional diseases**: functional obstructive bowel diseases, congenital or acquired aganglionosis, Ogilvie syndrome, megacolon, irritable bowel disease
- **Pelvic exit obstruction**: rectal prolapse, rectocele, rectal intussusception, rectal stenosis, megarectum, hypertonus of internal sphincter, paradoxical contraction of puborectal muscle

**Box 3. Causal and/or Contributing Factors to Constipation in Patients in Palliative Care (Modified from [20])**

**Organic Factors**

- **Pharmacological agents**: antacids, antiepileptics, antiemetics (5-HT3 antagonists), antihypertensives, antiparkinsonians, anticholinergics, antidepressants, antitussives, antidiarrheals (by causing dehydration), cancer chemotherapy agents, diuretics (by causing dehydration), iron (orally administered), opioid analgesics, neuroleptics
- **Metabolic abnormalities**: dehydration (fever, vomiting, polyuria, poor fluid intake, diuretics), hypercalcaemia, hypokalaemia, uraemia, hypothyroidism, diabetes
- **Neurological disorders**: spinal cord involvement, sacral nerve infiltration, autonomic failure (Parkinson disease, multiple sclerosis, motor neurone disease, diabetic neuropathy)
- **Painful anorectal conditions** (haemorrhoids, anal fissure, perianal abscess)

**Functional Factors**

- **Diet**: Poor appetite and low amounts of food intake, low-fibre diet, poor fluid intake
- **Environmental**: Lack of privacy, comfort, or assistance with toileting
- **Other factors**: Advanced age, inactivity, decreased mobility, bed-ridden patients, depression, sedation

## What Measures Could Prevent or Correct the Factors Exacerbating Chronic Constipation in Patients Receiving Palliative Care?

In patients receiving palliative care, the underlying causal factors for constipation are likely to be long-standing. The patient's bowel pattern needs to be carefully assessed, with a focus on looking for modifiable factors. For example, if a particular pharmacological agent is identified as a possible causative factor, it may be helpful to change the agent or the route of its administration. It is also helpful to anticipate the constipating effects of medications, such as opioids, and to provide laxatives prophylactically. Other concomitant or contributing factors such as electrolyte imbalances or metabolic/endocrine abnormalities should be identified and corrected. However, the underlying cause of constipation is often unavoidable and pharmacological treatment is often necessary [1].

**Figure 3:**
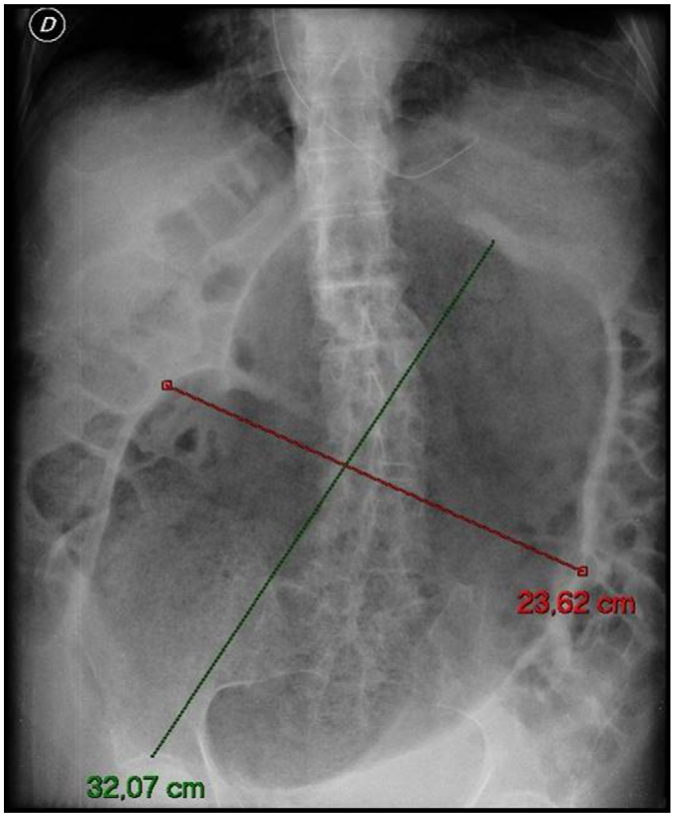
Plain abdominal X-ray after two days of medical treatment (rehydration, correction of electrolyte imbalance, and multiple enemas) showing enormous faecal impaction, although slightly reduced, still measuring 32 cm in length and 24 cm in width.

**Figure 4:**
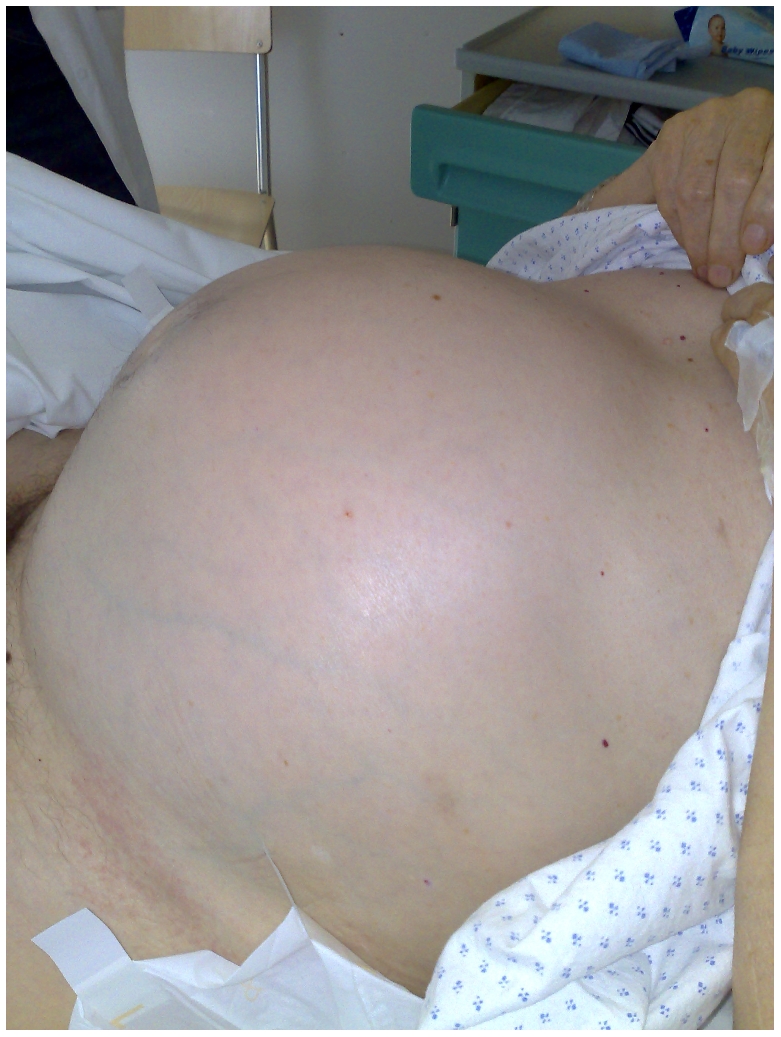
Clinical view of the abdomen, still grossly distended after ten days of conservative treatment.

In this patient, a long colonic-rectal tube was inserted with partial relief of the abdominal distension after expulsion of 8,000 ml of gas. The patient was admitted to the medical ward for rehydration, correction of electrolyte imbalance, and multiple enemas. Two days later the faecal impaction, although slightly reduced, still measured 32 cm in length and 24 cm in width (Figure 3). After ten days of conservative treatment, the abdomen remained visibly distended (Figure 4).

## Which Diagnostic Exam Would Now Be Helpful?

Serial plain abdominal X-rays had already been performed. Colonoscopy was not feasible, because the severe faecal impaction may have hindered the progression of the endoscope and affected the sensitivity of the exam by covering and camouflaging possible mucosal lesions of the colonic wall. Contrast enema would probably not have been effective or diagnostic. Therefore abdominal CT scan with multiplanar reconstruction and three-dimensional (3-D) reconstruction appeared to be the best option as a further diagnostic step, in order to assess the presence of an anatomic cause for the patient's bowel obstruction.

**Figure 5:**
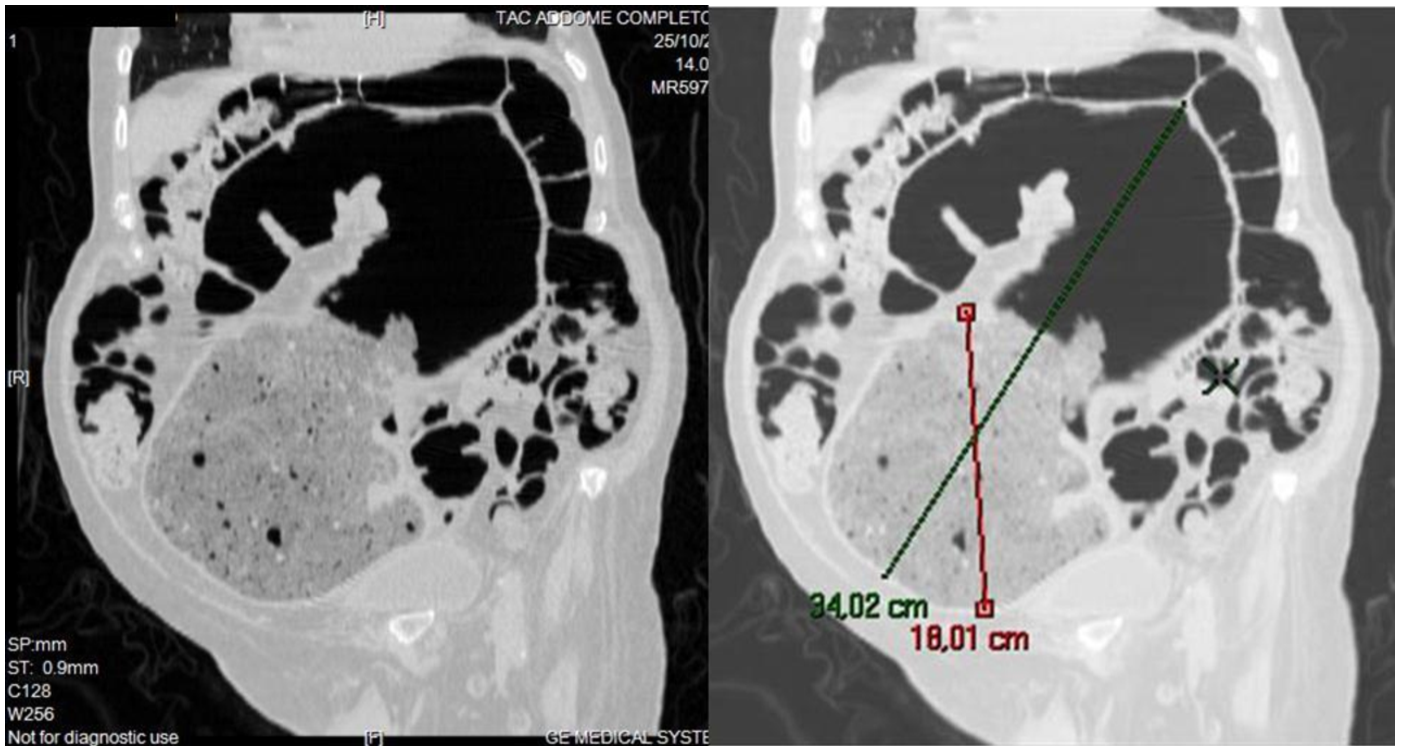
Abdominal CT scan with multiplanar reconstruction showing the persistence of a large faecal impaction, over 18 cm in extent, located in the sigmoid colon, as well as a massive dilatation of the colonic wall and the descending sigmoid colon forming a twisting loop.

Abdominal CT scan was ordered. The multiplanar and 3-D reconstruction showed the persistence of a large faecal impaction, over 18 cm in extent (Figure 5), in the sigmoid colon. There was massive dilatation of the colonic wall and twisting of the descending sigmoid colon.

**Figure 6:**
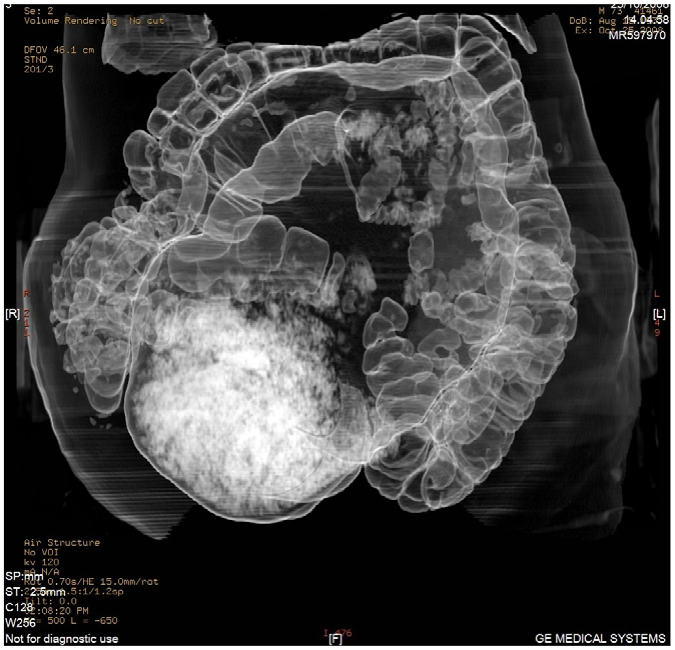
Multiplanar and 3-D reconstruction CT scan showed abnormally dilated descending colon and tortuous twisting loop, worsening the neurogenic chronic faecal stagnation.

It is likely that the abnormally dilated descending colon was exacerbating the neurogenic chronic faecal stagnation (Figure 6).

## What Was the Next Step in Managing This Condition?

Rehydration, multiple enemas, the prokinetic agent neostigmine (see Box 4), and laxatives were continued with gradual relief of the bowel obstruction and resolution of the faecal impaction.

**Box 4. Prokinetic Agents for Gastrointestinal Motility Disorders [21],[22]**

- **Cholinergic agents (bethanechol):** indications: postoperative ileus; limitations: side effects
- **Dopamine antagonists (domperidone):** indications: gastroparesis and gastroesophageal reflux; limitations: poorly effective in colonic motility disorders
- **Opioid antagonists (naloxone):** indications: irritable bowel syndrome, small intestinal pseudo-obstruction, constipation
- **Motilin agonists (erythromycin):** indications: diabetic gastroparesis, colonic pseudo-obstruction, postoperative ileus
- **Cholinergic agonist and dopamine antagonist (metoclopramide):** indications: exclusively for proximal motility dysfunction
- **Parasympathomimetics (cisapride):** indications: colonic motility disorders, constipation-predominant irritable bowel syndrome
- **Partial serotonin agonist (tegaserod):** indications: constipation-predominant irritable bowel syndrome; limitations: cardiovascular adverse effects
- **Acetylcholine esterase inhibitor (neostigmine)**: indications: colonic pseudo-obstruction
- **Prostaglandins:** lubiprostone (prostaglandin E1 derivative) and oral prostaglandin E2 are reported to increase small intestine and colonic transit
- **Cholecystokinin antagonist (ceruletide):** enhances gastrointestinal motility
- **Analogue of somatostatin (octreotide):** experimental evidence of shortening ileus and promoting bowel movements in the small intestine and colon in animal models

We believed that neostigmine, which has a focused effect in stimulating large bowel motility, was the most appropriate and beneficial prokinetic agent in this setting (it is also appropriate in patients with colonic pseudo-obstruction).

The patient was scheduled for elective surgical resection of the redundant megacolon. He was discharged 25 days after admission, after rehydration, multiple enemas, prokinetic agents, and laxatives. On discharge, the abdomen remained slightly distended but was soft and non-tender. The patient was passing stools and not vomiting. We suggested home nursing to the patient and his family.

After discharge, he underwent elective surgical resection of the redundant megacolon with planned primary anastomosis, and he had an uneventful postoperative course.

The patient gave written consent for these case details to be published.

## Discussion

If people in Western societies continue to live longer, we are likely to see an increase in the number of institutionalised elderly people with impaired mobility. Both ageing and immobility are risk factors for constipation. The estimated prevalence of constipation is between 2%–28%, and the number of people reporting constipation increases with age [2]–[4]. Constipation is more severe in those with pre-existing neurological illness and injury [5],[6]. A US study found that constipation was more common in women, African Americans, people from lower socioeconomic levels, and those living in rural areas and northern states [7]. Faecal impaction is common in frail ill elderly people or in people of any age if they have a neurologic impairment (e.g., spinal cord injury, stroke, multiple sclerosis, spina bifida).

The prevalence of constipation among patients in palliative care ranges from 32% to 87%, and it is particularly common in patients with end-stage cancer [8]–[10]. The combination of physical illness and hospitalisation may cause and/or worsen constipation. About 50% of patients admitted to hospices cite constipation as a main concern [11], and this is the third most common symptom after pain and anorexia in patients in hospices.

### Complications of Constipation

In severe constipation, patients are usually unable to pass much stool and may pass only small amounts of watery stool. They typically experience abdominal pain, discomfort and bloating, and may also lose their appetite. Some very ill older patients may have a change in behaviour and may develop fever.

In patients with constipation associated with a sigmoid redundant megacolon, colonic volvulus can develop, with possible progression to bowel wall ischaemia and perforation. Early surgical consultation and laparotomy are mandatory in such cases.

### Treatment of Constipation

Treatment of constipation, and of the most severe forms of faecal impaction, is multimodal [12]. If a large impaction is present, it may need to be broken up manually, using lubricated gloved fingers with patients lying on their left side [13]. Multiple enemas with sodium phosphate and soapsuds [14], or even with natural mixtures such as milk and molasses, can contribute to removing any leftover stool (a clinical trial of a milk and molasses enema for childhood constipation is underway in the US; see http://clinicaltrials.gov/ct2/show/NCT00467350). Fibres and laxatives can increase stool frequency and improve symptoms of constipation [15]. Pulsed irrigation of faecal impaction is also used for bowel management in patients with chronic constipation. It has been used primarily in patients with neuropathic bowel who have failed conservative therapy. Hospitalisation for rehydration and electrolyte imbalance correction is required for some patients, and in the most severe cases manual disimpaction under general anaesthesia is required [16].

Diagnostic assessment for colonic diseases is not routinely required. Such assessment is usually reserved for patients whose constipation is refractory to conservative treatment, or who have persistent severe faecal impaction, and/or who are found to have concomitant colonic obstructive disease.

Management of chronic constipation is shown in Box 5. The different levels and steps in managing chronic constipation should be focused on the different underlying conditions and needs of patients. Management is primarily conservative for patients with mild long-term constipation, in younger patients, and in those who are otherwise healthy and will adhere to a conservative regime. Medical treatment should be initiated for moderate-to-severe constipation, in the elderly, institutionalised, critically ill, or neurologically impaired, and in patients in palliative care and who are at the end of their lives. Surgical treatment should be reserved for selected patients (see Box 5) and/or constipation that is persistent and unresponsive to the other treatments.

**Box 5. Management of Chronic Constipation**

1. **Conservative (for mild constipation, younger patients, those who are otherwise healthy and will adhere to a conservative regime)**
  - lifestyle modification (adequate intake of dietary fibre and fluids, regular physical activity)
  - behavioural approaches (habit training, biofeedback)
  - bowel training
2. **Medical (for moderate-to-severe constipation, elderly, institutionalised, critically ill, or neurologically impaired patients, or patients in palliative care settings)**
  - **Oral laxatives**
    1. **Predominantly softening:**
      1. Faecal lubricants: liquid paraffin
      2. Bulk-forming laxatives: methylcellulose, polycarbophil, psyllium, ispagula
      3. Macrogols: polyethylene glycol and electrolytes
      4. Osmotic hyperosmolar laxatives: lactulose, sorbitol
      5. Emollient stool softeners and surfactants: docusate calcium, docusate sodium
      6. Saline laxatives: magnesium citrate, magnesium hydroxide, magnesium sulphate, sodium biphosphate
    2. **Combination laxatives:**
      1. Softener and stimulant: poloxamer and dantron
    3. **Predominantly peristalsis-stimulating:**
      1. Anthraquinones: senna, danthron, cascara sagrada
      2. Polyphenolics and other stimulant laxatives: bisacodyl, sodium picosulphate, castor oil
  - **Rectal laxatives**
    1. **Predominantly softening:**
      1. Faecal lubricants: arachis oil enema, docusate sodium enema
      2. Osmotic laxatives: glycerol suppository
      3. Saline laxatives: phosphate enema, sodium citrate enema
    2. **Predominantly stimulating:**
      1. Polyphenolics: bisacodyl suppository
  - **Prokinetics drugs (see** **Box 4** **)**
  - **Injection of botulinum toxin**
3. **Surgical (for selected patients)**
  - Repair of non-emptying rectoceles for patients with obstructed defecation
  - Subtotal colectomy with ileorectal anastomosis for patients with persistent and intractable slow transit constipation
  - Patients with combined slow transit constipation and pelvic outlet obstruction, as well as symptomatic refractory retaining rectoceles and rectal intussusception, benefit from subtotal colectomy with ileorectal anastomosis and repair or treatment of the outlet obstruction causing pathology

### Managing Constipation in Palliative Care

In palliative care, nursing staff play a crucial role in the assessment, prophylaxis, and management of constipation, since they are in daily contact with patients. Such assessments should focus not only on the frequency of bowel movements, but also on the quality of stools; length of time to defecation; diarrhoea and overflow diarrhoea; continence and incontinence; effectiveness of laxatives; addition of complementary therapies worsening constipation; diet and fluid intake; environmental factors that could be influencing bowel movements (i.e., comfort and privacy); and the need for abdominal massage. Abdominal massage, performed by a massage therapist, may be used in patients with chronic constipation and altered motility as an adjunctive measure; it may help to stimulate the periphery of the small and large intestines and relieve bowel atony. A recent randomised controlled trial of abdominal massage plus laxatives versus laxatives alone for constipation found that the addition of massage was associated with decreased constipation and abdominal pain and increased bowel frequency [17].

Larkin and colleagues recently published an algorithm on prophylaxis, ongoing assessment, and treatment of constipation in palliative care settings [1]. Ongoing monitoring for early symptoms of constipation and patient education are the cornerstones of prophylaxis. The first step in treatment should be a careful assessment to confirm constipation and exclude malignant causes of intestinal obstruction. The next step is the identification and treatment of correctable causes. If the cause is not correctable, first-line treatment should be with oral laxatives (a combination of softener and stimulants according to patient needs [1]). If this treatment improves symptoms, it should be continued; otherwise a second-line treatment should be adopted. Standard second-line treatment is a rectal suppository and enema. If first-line and second-line treatments fail, the third-line treatment is manual evacuation. During second-line and third-line treatment, adding a peripheral opioid antagonist may be helpful if the patient is taking opioids.

**Key Learning Points**

- Older patients, institutionalised elderly people, and chronically bed-ridden patients commonly experience constipation and are at risk of developing severe fecal impaction.
- This risk is higher in the presence of neurological illnesses or injuries.
- The management is usually conservative and multimodal.
- The combination of a softener and stimulant laxative is generally recommended, and the choice of laxatives should be made on an individual basis.
- Diagnostic assessment for colonic diseases and surgical consultation are not routinely required.
- Unsuccessful conservative treatment, the persistence of severe faecal impaction, and/or the finding of concomitant colonic obstructive disease should lead to further diagnostic assessment and surgical consultation.
- Early surgical consultation and urgent laparotomy are required in the case of free air on plain abdominal X-ray and/or signs of peritonism and acute abdomen.

## Abbreviations

CT: computed tomography
